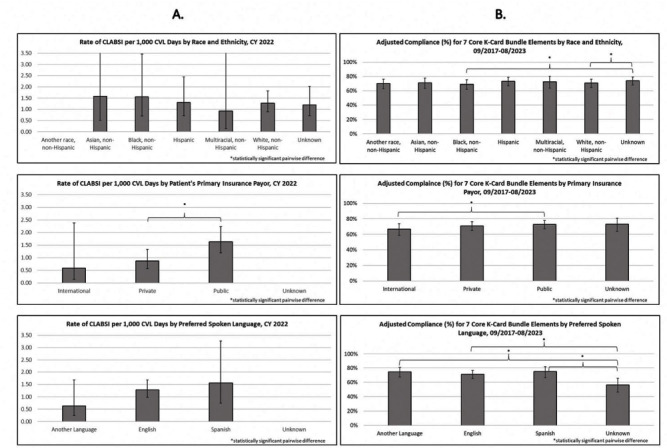# Iterative Health Equity Analyses of Central Line-Associated Bloodstream Infection (CLABSI) Events at a Pediatric Hospital

**DOI:** 10.1017/ash.2024.98

**Published:** 2024-09-16

**Authors:** Ana Vaughan-Malloy, Phillip Hahn, Paula Lamagna, Jenny Chan Yuen, Dionne Graham, Jennifer Ormsby

**Affiliations:** Boston Children’s Hospital; Boston Children’s Hospital, Program for Patient Safety and Quality

## Abstract

**Background:** Per the Centers for Disease Control and Prevention, health equity stipulates all have a fair, just opportunity to attain their highest level of health. Limited evidence exists for disparities in health equity and healthcare-associated infections (HAI), with no evidence on language or primary insurance payor. While reviewing quality metrics, a disparity signal in central line-associated bloodstream infections (CLABSIs) prompted a multidisciplinary deep dive, with iterative analyses to understand potential inequities to identify improvement opportunities. **Methods:** CLABSI data was stratified and analyzed for evidence of disparity by race/ethnicity, primary insurance payor, and preferred spoken language utilizing an internal methodology. Subsequent analyses included a root cause analysis (RCA), case mix index (CMI) analysis, analysis of CLABSI Kamishibai card (K-card) rounding to monitor maintenance bundle reliability, and comparison of distribution of central venous catheter (CVC) line days to K-card audits [Figure 1]. Chi-square tests were used to test for significant differences for categorical variables in RCA and K-card analyses. ANOVA was used to compare CMI between demographic groups. Multiple logistic regression was used to compare K-card compliance rates by demographic groups. **Results:** When stratifying CLABSI rate by primary payor, pairwise comparisons indicated patients with a public payor had a statistically higher rate of CLABSI compared to private (p=0.02) [Figure 2A]. RCA analysis revealed when compared to patients with private payors, those with public had significantly higher rates of overdue needless connector changes (p = 0.03) and increased number of daily CVC entries (p = 0.05), while patients speaking another language (p = 0.02) were significantly more likely to have CVC contamination events. CMI analyses on CLABSI cases did not show patient acuity to vary significantly between demographics. Bivariate analysis of K-card data revealed minor differences in reliability with 7 Core Maintenance Bundle Elements by demographics; adjusting for all demographics and accounting for unit, pairwise comparisons indicated public payors had significantly higher compliance than international [Figure 2B]. We found no major differences in demographic distribution of CVC line days compared to K-card audits, suggesting we representatively audit maintenance bundle process measures. **Conclusions:** Our review of health equity in CLABSI events ultimately led to subsequent questions requiring analysis of other data sets. Utilizing an exploratory approach and assembling a multidisciplinary team to identify potential drivers of identified disparities adds value to health equity analyses. This is the first description of HAI data beyond race and ethnicity and can assist other institutions in their process of evaluating healthcare disparities and HAIs.

**Figure:**
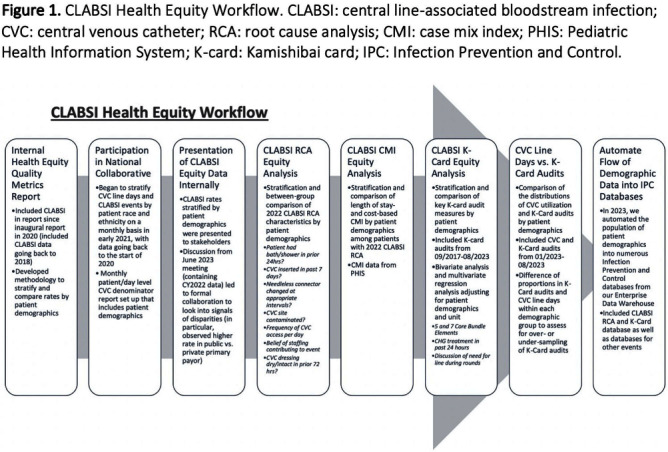

**Figure:**